# 小于30岁低龄肺癌患者的临床分析

**DOI:** 10.3779/j.issn.1009-3419.2011.05.14

**Published:** 2011-05-20

**Authors:** 广杰 侯, 连斌 张, 向阳 初, 继云 王

**Affiliations:** 1 450003 郑州，河南省人民医院胸外科 Departmentof Thoracic Surgery, the People's Hospital of Henan Province, Zhengzhou 450003, China; 2 100853 北京，中国人民解放军总医院胸外科 Departmentof Thoracic Surgery, the General Hospital of the People's Liberation Army, Beijing 100853, China; 3 065000 廊坊，中国石油天然气集团公司中心医院胸外科 Departmentof Thoracic Surgery, the Central Hospital of China National Petroleum Corporation, Langfang 065000, China

**Keywords:** 肺癌, 病理, 低龄患者, Lung neoplasms, Pathology, Young adult

## Abstract

**背景与目的:**

青年肺癌患者肿瘤恶性程度高，侵袭性强，预后差，已成为多数共识。既往对青年肺癌患者的研究多以40岁或45岁为界，而 < 30岁的低龄肺癌患者临床资料少见报道。本文回顾分析了解放军总医院从1993年至今17年来诊疗过的 < 30岁的低龄肺癌患者的病史、分期、治疗及病理特点，为这一年龄段肺癌患者的诊疗提供参考。

**方法:**

检索解放军总医院收治的1993年以来所有 < 30岁的肺癌患者，共计53例。其中非小细胞癌患者34例，小细胞癌患者19例。男女比例1.5:1。非小细胞肺癌患者中，腺癌27例，鳞癌6例，腺鳞癌1例，无大细胞癌患者。其中12例接受手术治疗，38例化放疗，3例放弃治疗。

**结果:**

全组无住院死亡病例，12例手术患者中，手术根治性切除8例，姑息性切除4例。

**结论:**

低龄肺癌患者腺癌，小细胞癌比例大，多数出现症状就诊时处于晚期，预后差。改善预后应重视常规体检，早期诊断。

## Abstract (en)

**Background and objective:**

It is common recognized that young patients of lung cancer have poor prognosis due to relatively higher malignancy and more invasive growth. In the past most studies on young patients of lung cancer selected patients younger than 40 or 45 years old, and there were few clinical materials for younger patients under 30 years. This study retrospectively described the the disease history, stage, treatment and pathology features of lung cancer patients younger than 30 years and aimed to provide references for these patients.

**Methods:**

Those patients younger than 30 years, once admitted in the General Hospital of the People's Liberation Army for lung cancer from 1993 to date, were sought in medical record system, and 53 patients were found in total. In this group, there were 34 non-small cell lung cancer (NSCLC) patients and 19 small cell lung cacer (SCLC) patients. The male/female ratio was 1.5:1. In the NSCLC patients, there were 27 adenocarcinomas, 6 squamous carcinomas and 1 adenosquamous carcinoma, with no large cell carcinoma involved. In these patients, 12 patients received operations while 38 patients got chemo- and/or radiotherapy and 3 quited any treatment.

**Results:**

There was no death in hospital, however, in the 12 patients who got operation, only 8 patients got complete resection while 4 patients got palliative resection.

**Conclusion:**

Lung cancer patients younger than 30 years had a high fraction of adenocarcinoma and small cell type pathologically and most of them were in late stage when presenting with symptoms in hospital and would have a dismal prognosis. The routine health examination and early diagnosis should be emphasized to improve the prognosis of these patients.

年轻人中肺癌发生率很低，既往对青年肺癌研究多以40岁或45岁为界，而对于 < 30岁的人群肺癌的概况少有文献报道^[1, 2]^。作者检索了解放军总医院从1993年至今年龄 < 30岁的肺癌患者的临床资料，总结如下。

## 1 材料与方法

### 1.1 临床资料

采用解放军总医院病案检索系统，检索所有诊断为原发肺腺癌、鳞癌、大细胞癌、小细胞癌的年龄 < 30岁的患者，涵盖呼吸科，肿瘤科，胸外科等相关科室，共检出53例。分布科室：呼吸科22例，肿瘤内科11例，胸外科17例，其它科3例。患者入院时间自1993年至今。

53例患者中，男性32例，女性21例，男女比例1.5:1。 < 20岁的患者6例，占11.32%，年龄最小者15岁。患者均常规行影像、气管镜等检查。所有患者均取得病理诊断（气管镜或手术），按照病理类型分类：非小细胞肺癌34例，占64.15%，其中腺癌27例（50.94%），鳞癌6例（11.32%），腺鳞癌1例，无大细胞癌。小细胞癌19例，占35.85%。

主要初始症状体征包括胸痛、胸闷、声嘶、颈部锁骨上窝肿块等。患者以肺内多发转移最为常见（8例），其次为脑转移5例，骨转移3例，肝转移2例，合并恶性胸腔积液6例，颈部锁骨上淋巴结肿大6例，上腔静脉综合征2例。腺癌患者肺内转移或/和胸膜转移胸腔积液最为常见，而小细胞肺癌患者肺门部巨大肿块伴有纵隔淋巴结肿大融合及多发脏器转移。

回顾病史，共9例患者吸烟，均为男性，吸烟率16.98%。其中，腺癌患者27例中吸烟者2例。小细胞癌患者19例中吸烟者7例，最重者40支/天，烟龄10年。鳞癌患者6人中无吸烟者。

### 1.2 治疗

肿瘤科、呼吸科等科室共36例患者均在治疗前明确病理诊断，均为临床分期Ⅲb期或Ⅳ期的非小细胞肺癌或小细胞癌，1例放弃治疗，35例实行化疗或联合化放疗。

胸外科的17例患者中，2例放弃治疗，3例化放疗，12例实施手术，手术率22.64%（12/53）。其中，开胸探查术2例，全肺切除4例（包括1例全胸膜肺切除），肺叶切或中下叶切除6例。完全手术切除（R0, no residual tumor）8例，姑息性手术（R1, microscopic residual tumor or R2, macroscopic residual tumor）4例，占33.33%。术后分期Ⅰ期5例，Ⅱ期2例，Ⅲa期1例，余4例为Ⅲb期或Ⅳ期。术后病理：腺癌8例，小细胞癌2例，鳞癌2例。

## 2 结果

12例手术患者中，1例小细胞癌患者右全肺切除术后当天发生股动脉栓塞，行股动脉切开取栓术后痊愈出院，余11例无严重并发症。无手术死亡患者。

特殊病例：罗某，女，16岁。因喉气管多发乳头状瘤行气管切开，气管内镜下治疗10余次，因诊断左肺感染，呼吸衰竭入监护室治疗，经气管镜检查诊断为左肺中分化鳞癌合并感染，患者感染控制后出院。

患者总体分期，Ⅰ期5例，Ⅱ期2例，Ⅲa期1例，Ⅲb期或Ⅳ期26例，小细胞癌局限期5例，广泛期14例。

全组53例出院评价好转39例，治愈8例（手术患者），无效2例，未治3例，其他1例。全组无住院死亡病例。

仅3例非手术患者有再次或多次住院记录，接受化放疗，但1年-2年内病变均进展，广泛转移。本组患者时间跨度较大，失访率高，随访不完善。

## 3 讨论

低龄（< 30岁）肺癌临床较为罕见，既往对年轻肺癌患者的临床研究多以40岁或45岁为界，且多为外科或内科的单个科室的经验总结，不能全面反映低龄肺癌患者的临床特点^[1, 2]^，而对于 < 30岁的低龄肺癌患者则少有相关的临床资料总结。本文通过回顾总结，全面反映这一少见患者群体的发病及诊疗状况。作者检索了解放军总医院所有诊断为原发支气管肺癌的 < 30岁患者的临床资料，患者时间跨度17年，涵盖胸外科、肿瘤科、呼吸科等相关科室，可以基本全面反映这一年龄段患者的临床特点，但由于患者时间跨度大，发病时多为晚期，生存时间短，因此多数无法随访，是本研究的缺憾。

从发病率来说，作者检索同期45岁以下原发肺癌患者，发现30岁以下患者约占10%左右，而45岁以下患者约占肺癌患者总数的10%左右。这一比例与另一以上海为中心的大组病例研究基本相符。可以基本反映国内低龄肺癌患者的构成比^[3]^，即30岁以下低龄肺癌患者，约占肺癌人群总数的1%左右。

低龄患者病理类型以腺癌，小细胞癌为主，而鳞癌比例较低，这一病理特点与多数文献^[3, 4]^报道相符。值得注意的是，在检索过程中，作者还检出7例支气管腺体肿瘤肿瘤。支气管腺体肿瘤不同于肺癌常见的四大病理类型，它起源于支气管表皮细胞下的腺体，包括类癌、腺样囊性癌、粘液表皮样癌等，约占总体肺癌数的2%-6%。其恶性程度低，好发于气管及近端支气管，预后相对较好。因其具有不同的临床特点，作者将另文总结。

较其他年龄段患者，青年肺癌分化差，侵袭性强，就诊时多为晚期，总体预后差已经得到多个研究的证实^[3, 4]^。本组绝大多数患者就诊时处于Ⅲb期或Ⅳ期，失去手术根治机会。即使手术，其探查或姑息切除比例也较高，本组姑息切除率高达33.33%。究其原因，影像分辨率差影响手术评估是主要原因（主要发生在早年），也有医生主观准备不足的可能。低龄患者往往以胸闷、疼痛、声嘶、颈部锁骨上淋巴结肿大等转移症状就诊，姑息性治疗成为主要治疗模式，控制恶性胸水，减轻疼痛和压迫症状成为主要治疗内容。对于一部分可以手术治疗的患者，务必在术前完善检查，获得病理诊断，全面评估病情，准确分期，使得患者能从手术中获益。

相比同分期的老龄患者，青年患者对手术、化放疗具有更好的耐受性，采用更积极的联合治疗包括靶向治疗可能使之获得较好预后^[5, 6]^。但从总体来看，由于就诊时多处于晚期，只有很少数患者可以得到根治性的治疗，大多数患者只能减轻症状，延长生存期。面对这一年轻的患者群，除常规的治疗内容以外，如何对患者个人、家庭进行心理疏导也值得医务人员思考。

低龄肺癌患者容易发生误诊或漏诊。当影像表现为肺部孤立结节或占位时，临床上误诊为肺炎，结核的情况经常发生^[5]^。原因主要是这一人群肺癌发病率低，往往不被重视。回顾本组患者病史，有5例患者外院初诊时误诊而接受过抗炎抗结核治疗，有的时间长达半年，直至病变明显进展。作者深刻体会到，在肺疾病的诊断中，积极的有创检查是非常重要的。对于那些难以确诊的患者，或短期抗感染抗结核治疗无效的患者，积极的有创检查如气管镜、肺穿刺，甚至胸腔镜检查可能使患者获得更快诊断中从而受益。

如何改善低龄肺癌患者的总体预后？常规体检早期发现无疑是最有效的方法，本组有5例Ⅰ期肺癌，有望术后获得长期生存，均为体检偶然发现的。在实际生活中，年轻人常自恃身体好，不重视自身健康，且多无固定工作，体检往往被忽略或走过场，待出现症状为时已晚，这是造成低龄肺癌患者多处于疾病晚期的重要原因。因此，改善低龄肺癌患者的预后，也要从提高年轻人健康意识，重视常规体检着手。
